# Promoting caregiver involvement at the public library: An evaluation of a math and science storytime program for young children

**DOI:** 10.3389/fpsyg.2022.1049694

**Published:** 2022-12-07

**Authors:** Larissa Gaias, Michelle Taylor, Megan E. Pratt, Mariko Whelan

**Affiliations:** ^1^Department of Psychology, University of Massachusetts Lowell, Lowell, MA, United States; ^2^Department of Family and Consumer Sciences, California State University, Long Beach, Long Beach, CA, United States; ^3^Oregon Child Care Research Partnership, Oregon State University, Corvallis, OR, United States; ^4^Scottsdale Public Library, Scottsdale, AZ, United States

**Keywords:** community program evaluation, public libraries, early childhood, school readiness, STEM education, parenting program, parent–child interactions, static group comparison design

## Abstract

**Introduction:**

Public libraries are asset institutions that provide important spaces for families to engage in meaningful, authentic STEM learning. However, limited budgets and a model centered on open-access and broad inclusion makes conducting rigorous evaluations in these spaces, such as randomized control trials, challenging. There is a need to consider evaluation designs that consider both rigor and feasibility. The aims of the present study were to: (1) describe an innovative interactive parent–child interactive storytime program, Fun with Math and Science (FMS); and (2) conduct a preliminary evaluation of FMS in a large, urban public library setting, using a quasi-experimental static group comparison design.

**Methods and Results:**

Post-test scores for caregivers who completed the program in the fall or winter (n = 80) were compared to pre-test scores for caregivers who completed the program the following spring (n = 35); Fall/winter caregivers scored higher on program items related to concrete behaviors to support math and science learning, but significant differences were not found on items related to caregiving beliefs or general caregiving practices. Demographic differences were also found related to program outcomes.

**Discussion:**

Results are discussed both in terms of implications for the development and implementation of caregiver-child interactive programming, as well as the use of innovative analytic approaches to program evaluation in community settings.

## Introduction

Children’s school readiness skills at formal school entry have been shown to predict children’s academic trajectories, with math holding the greatest predictive power of later achievement (Duncan et al., 2007). Unfortunately, many children enter school struggling with the underlying skills important for later math and science achievement. With the growing awareness of the importance of these skills as a part of promoting children’s STEM (Science, Technology, Engineering, and Math) achievement, along with the increased attention to the importance of investing in the early childhood years, efforts are being made to more strongly and deliberately incorporate early math and science programming into early learning settings (Brenneman et al., 2009; Hassinger-Das et al., 2020). Much of this attention has been given to formal early childhood education (ECE) settings, such as center-based preschool programs (Clements and Sarama, 2011; Kermani and Aldemir, 2015), with less attention to programming targeting caregivers’ capacities to promote their young child’s emerging math and science skills. To support early learning within families, there is a movement underway as informal community settings, such as libraries and museums, strive to become more interconnected with the early learning frameworks of their communities (Institute of Museum and Library Services, 2013; Families and Work Institute, 2015). This movement highlights the role of caregivers as a child’s first teacher, encouraging libraries and other informal institutions to create experiences that target not only the child, but also teach caregivers how to effectively engage in their children’s learning. The current study investigates a public library program designed to teach caregivers how to support their preschool-age child’s math and science skills using an interactive, storytime format. Specifically, the study addresses the potential for interactive family involvement programming to promote positive caregiving practices and attitudes important for supporting early math and science learning.

### Role of caregivers in young children’s math and science learning

Caregivers’ expectations for their children predict children’s later attitudes about and achievement in math and science domains (Parsons et al., 1982; Crowley et al., 2001; Kleemans et al., 2012; Skwarchuk et al., 2014). For example, in one study, students who perceived support from their parents in math and science concepts tended to feel more efficacious and have positive attitudes towards math and science (Rice et al., 2013). In contrast, Tenenbaum and Leaper (2003) found that mothers who believed their children found science difficult and boring had children who were more likely to report poor ability and low interest in science. Although most of this research is with older children, there is some evidence that expectations are important for younger children as well; indeed, one study found that parents’ numeracy expectations about what their preschool child should know predicted children’s early numeracy outcomes at the end of kindergarten (Kleemans et al., 2012).

In addition to caregivers’ perceptions of what young children should know related to math and science, how caregivers and children spend time together also matters. For example, when caregivers provide math and science activities at home, children’s early skills in these areas improve (Kleemans et al., 2012; Skwarchuk et al., 2014; Hart et al., 2016; Daucourt et al., 2021). The work by Kleemans et al. (2012) found that the presence of numeracy activities predicted children’s early math skills at the end of kindergarten, above and beyond parent’s expectations. Hart et al. (2016) found that parents who reported doing more math activities in the home reported having children with higher math skills; importantly, parent’s own anxiety about math was not a significant predictor of child’s skills. Further, Skwarchuk et al. (2014) found that both prekindergarten formal (e.g., practicing simple sums) and informal learning (e.g., games with numbers) activities in the home environment predicted children’s numeracy outcomes. In fact, a recent meta-analysis conducted by Daucourt et al. (2021) found that the home math environment (including math-activities, beliefs, attitudes, expectation, and interactions) is significantly associated with children’s math achievement. Similar results have been shown regarding science learning as well. One recent study demonstrated that preschool aged children who were exposed to science interactions and learning opportunities in the home, including both science content and engineering practice, demonstrated higher levels of science core knowledge (Westerberg et al., 2022). Similarly, Junge et al. (2021) found that parental engagement in science-related activities is associated with preschool children’s science knowledge. Engagement in these science activities fully mediated the relationship between parental level of education, parents’ interest in science, and home language on child’s science knowledge, controlling for children’s overall cognitive abilities and gender. Together, these studies emphasize the importance of caregivers’ attitudes, beliefs, and practices related to early math and science learning for fostering young children’s STEM knowledge and skills. Thus, programs that enhance caregivers’ ability to build strong science and math home environments for their children will likely have positive and meaningful impacts on children’s early learning.

### Promoting positive caregiving related to math and science

With the growing awareness of the value of caregivers in supporting math and science learning among young children for later school success, a burgeoning set of intervention programs for caregivers of young children have emerged, providing preliminary evidence that intervention efforts in both the home and at school can significantly improve caregiving practices and, ultimately, promote children’s emerging math skills (Starkey and Klein, 2000; Berkowitz et al., 2015). For example, one randomized trial found that when parents of young children engaged with a mobile-device app program designed to promote math through short numerical story problems during bedtime routines, children performed significantly better on math achievement across the school year, particularly among children whose parents were anxious about math (Berkowitz et al., 2015). In another study, a Head-Start preschool based program designed to engage parents and children together in math learning through biweekly class sessions found that, parents who engaged in the program were better able to support children’s math learning than those in the control group (Starkey and Klein, 2000).

In contrast to early math, there is a dearth of research on efforts to promote parent–child learning related to early science skills (see Salvatierra and Cabello, 2022 for a review), which may have to do with a common perception that, compared to other school subjects, science learning is for older children (Andre et al., 1999). An exception is evidence of a children’s museum based intervention that found that providing families with enhanced family interactions (e.g., elaborative questions that prompt science thinking) appear to increase parents’ ability to support young children’s STEM learning (Haden et al., 2014). The current study builds on past work by investigating the potential for a public library math and science program to promote early math and science skills by targeting both parents and children.

### The public library as a place for math and science learning

Community settings, like museums and libraries, encourage family involvement in a child’s learning through shared interactive experiences. Acting as informal learning settings, these institutions are designed to promote rich conversation and teaching opportunities, and thus hold great potential as settings well positioned to promote caregiver engagement and teach caregivers best practices around supporting their young children’s learning of skills that will set a foundation for long-term achievement (see Tenenbaum et al., 2005; Haden, 2010). These caregiver-targeted efforts are largely underway in science and children’s museums (Families and Work Institute, 2015) that encourage hands-on learning and shared experiences; however, museums do not exist in every community and are often cost prohibitive because of admission fees. In contrast, public libraries exist in nearly every U.S. community and are characterized as welcoming, no (or very low) cost institutions. It should be noted that disparities do exist in both who accesses and is represented in library settings and materials, due to the pervasiveness of White, middle-upper class norms that are also reflected within our society at-large (Honma, 2005; Gibson et al., 2017; Schlesselman-Tarango, 2017). Although there is an indicated need for more explicit attention toward social justice in these settings, libraries continue to provide a critical role as community anchors. Notably, in recent years, these spaces have shifted their focus from what they can do *for* people to what they can do *with* people, resulting in greater attention to the experiences within the library, including early learning programs (American Library Association, 2015; Clark, 2017).

The experiences, resources, and interactions provided by public libraries fuel a love of learning. The Pew Research Center reports that the majority of parents of young children, especially families who earn less than $50,000, believe that libraries are “very important” for their children, and are interested in more and varied family library services, such as programming (Zickuhr et al., 2013). In response, public libraries continue to evolve to the needs and interests of their communities by tailoring their service model to provide more educational programming in addition to their traditional role of providing information to people, largely through book lending (Ralli and Payne, 2016; Lopez et al., 2017). Early childhood has become an increased focus of public libraries. Indeed, a seminal report called on libraries and museums to strive to provide high quality learning opportunities for young children, arguing that they are essential community resources that are ideal for supporting children’s school readiness and caregiver involvement (Institute of Museum and Library Services, 2013). As one example, Play-and-Learn spaces were developed in collaboration between librarians, developmental scientists, and architects to build physical environments within a library (e.g., climbing walls with letters that children can follow to create words) to encourage learning, discourse, and playful interactions (Hassinger-Das et al., 2020). These spaces have been associated with promotive caregiver-child interaction and conversation that can facilitate STEM learning (Hassinger-Das et al., 2020). Providing space for interactive and unstructured play with educational materials is a cornerstone of such programs, allowing children to explore their environments, interact with adults and peers, and grow their love for learning (Gray et al., 2022). Other research has emphasized the importance of using library storytime programs to enhance children’s learning. Although historically much of this work has focused on the importance of storytime for enhancing literacy skills (Albright et al., 2009; Campana et al., 2016), Campana (2020) found that librarians were incorporating numeracy and other early math content and skills naturally into storytime programs and that children were demonstrating math behaviors and knowledge during storytime activities. Related to the findings by Hassinger-Das et al. (2020) and Gray et al. (2022) discussed above, Campana et al. (2022) have emphasized the importance of incorporating more in-depth playful learning experience into the traditional library storytime for increasing children’s learning behaviors. Research has also shown that parents are drawn to library storytimes for the playful activities and opportunities for interaction (Cahill et al., 2020).

In addition, *enhanced storytime programs* build upon a traditional storytime format, where a librarian reads books and sings songs for a group of children, to pause and talk directly to the adults to teach caregivers tips and strategies. The most well-known enhanced storytime program is Every Child Ready to Read (ECRR), a joint venture undertaken by the Association for Library Services to Children and the Public Library Association. During program sessions, caregivers are led through activities with their children that promote early literacy skills and are taught how to apply and expand on these learning strategies in their day-to-day interactions with children at home. Evaluation results of the ECRR program indicate that the enhanced storytime format does in fact promote family engagement (Neuman et al., 2017), with parents demonstrating an increased understanding of literacy and motivation to support emerging literacy skills in their children (Stewart et al., 2014). Further, another study found that after the program, parents increased both their use of effective literacy practices and perception of the library as a resource for child learning (Neuman and Celano, 2007). A recent study on another enhanced storytime program that incorporates both parent education tips and caregiver-child interactive play focused on social–emotional development and literacy into a traditional storytime library program, called Books Can…^©^ (Blinded for review), also demonstrated promise for enhancing parents’ knowledge, attitudes, and behaviors (Blinded for review). These evaluations provide initial evidence that enhanced storytime programs can promote caregiving practices that encourage early learning.

Interactive caregiving programs also have the potential to enhance positive practices more broadly. As caregivers practice strategies to support their child’s early learning, they also likely increase their beliefs, practices, and perceptions of self-efficacy regarding engaging in positive caregiving practices (Welsh et al., 2014); in fact, multiple parenting interventions that target various domains of early learning have also found impacts on broader parenting outcomes, including parenting self-efficacy, child-directed interactions, and relationship quality (Wagner and Clayton, 1999; Pelletier and Brent, 2002). The current study focuses on how a math and science focused enhanced storytime program improves caregiving knowledge, beliefs, and practices related to this domain, as well as positive caregiving more generally.

### The program: Fun with Math & Science^©^

Fun with Math & Science^©^ (FMS; blinded for review) is a 6-week enhanced storytime program for caregivers and their preschoolers delivered by trained library staff. Guided by the National Association for the Education of Young Children’s Developmentally Appropriate Practices, it takes a progressive approach to education focusing on multicultural education, constructivism, and child-centered curriculum (National Association for the Education of Young Children, 2009). Through interactive parent–child class sessions, the program aims to improve caregiving beliefs, knowledge, and practices known to promote children’s early math, science, and literacy skills. The initial program developed by library staff was rewritten in 2015 by the library’s early learning coordinator to align with the state of Arizona’s Early Learning Standards for Math and Science and Arizona’s School Readiness Framework (Arizona Department of Education, 2013). Through the Partnership for Family-Library Engagement (blinded for review), the authors of this paper were then asked to partner with the library to further refine the program to ensure research-based best practices related to child development and parent engagement were utilized. Each 45-min session covers a different math or science topic, including: using your senses, counting and comparing, geometry and identifying attributes, sorting and classifying, patterning/sequencing/making observations, and measurement/hypothesizing/experimenting. Each session includes: an introduction to the concept of the week, sharing of four practical caregiving tips, interactive adult-child activities, book reading, and active songs. In addition, sessions focus on teaching, modeling, and practicing new skills. Specifically, (a) caregivers are explicitly *taught* current child development information and developmentally appropriate caregiving strategies; (b) instructors *model* the quality adult-child interactions during the course; and (c) time and space is provided for caregivers to immediately *practice* these new skills with their child during activities. After each session, children are given a book and caregivers are given a tip sheet to take home.

### Assessing program effectiveness in real-world community settings

Funding for community-based programs continues to prioritize “evidence-based” programming, making evaluations of programs such as FMS^©^ a priority. Pretest-posttest designs are commonly used in community-based research to assess change resulting from participating in a program or other intervention effort, despite their vulnerability to threats of internal (i.e., the degree to which the program causes change in the study sample) and external (i.e., the degree to which the program effect can be generalized to other populations and settings) validity. Rather, this design is used because it is relatively more feasible and requires fewer resources and demands than more rigorous designs that employ a control group. For example, a randomized control trial (RCT) design, often regarded as the gold standard, can best isolate program effects and protect against threats to validity, especially internal validity. However, for many community-based institutions, such as public libraries, limited and fluctuating yearly budgets prohibit rigorous evaluation, including other quasi-experimental designs that use a recruited comparison group (e.g., matched pairs). In addition, because libraries are inclusive community hubs that provide access to programming for all users, limiting service delivery to some families and not others can be unethical.

As such, it is important to consider innovative ways to utilize pre-post data that can increase understanding related to the effectiveness of community programs, because this design is commonly used and is sensitive to ethical concerns and issues of feasibility. The current study addresses this challenge by utilizing multiple waves of pre-post data in an innovative way. Specifically, we conduct a pre-experimental static group comparison design whereby we compare the *post-survey* of caregivers who participated in the program during an earlier time point (fall/winter) to the *pre-survey* of caregivers participating in the program at a later time point (spring). Using this design allows for the latter group to serve as a non-random control group for examining the relationship between program participation and measurable outcomes (Shadish et al., 2001). Importantly, because the control group was drawn from the study sample itself rather than the general public, we have increased confidence that the two groups are comparable. This method has been used in recent evaluations of community-based programs with similar goals, structures, and constraints on implementation (Andrews et al., 2020).

### Present study

The present study employs a static-group comparison design to investigate the potential effectiveness of the public-library-based Fun with Math & Science^©^ program on caregiving outcomes among a sample of families with preschool-age children. Specifically, the study asks:

RQ1: Does participating in FMS improve caregiving knowledge, beliefs, and practices related to math and science, when controlling for family demographic characteristics?

RQ2: Does participating in FMS improve positive caregiving generally (i.e., parenting behaviors, self-efficacy, progressive parenting beliefs), when controlling for family demographic characteristics?

Results of this study have implications for informal learning and community-based efforts to promote school readiness skills for children and supporting caregivers as a child’s first teacher. This study also provides a framework for other researchers who, because of practical real-world constraints, are unable to employ resource-heavy experimental design strategies to evaluate a community-based program or service.

## Materials and methods

### Participants and procedures

A total of 115 families participated in the Fun with Math & Science^©^ program, 80 in the Fall/Winter season and 35 in the Spring season. Demographic characteristics for participating families can be found in Table 1. Data for this study were collected in conjunction with the administration of the FMS programming, which was delivered according to regular library scheduling. The programs were offered in the fall, winter, and spring of the 2015–2016 calendar year at five different library locations and one community center within a single library system. In total, there were 13 different offerings of the 6-week program. For every week that a family attended the program, their child received a book to take home, and parents received a tip sheet relevant to the content for that session. Additionally, in order to encourage participants to attend all 6 weeks of the program, children received an incentive (a small backpack with a science journal, and math and science tools such as a measuring tape, magnifying glass, magnetic wand, bug catcher and eye dropper) if they attended at least five of the six sessions.

**Table 1:** Participant demographics.

	*N*	*%*
Total	115	100
Caregiver role		
Mothers	92	80
Fathers	11	9.6
Grandmothers	6	5.2
Other	5	4.4
Race/ethnicity		
White	69	60
Hispanic/latinx	21	18.3
Asian Indian	18	15.7
East Asian	7	6.1
Black	3	2.6
Middle Eastern	2	1.7
Native American	2	1.7
Home language		
English	98	85.2
Spanish	16	13.9
Other	22	19.1
Survey language		
English	113	99.1
Spanish	1	0.1
Highest level of education		
Did not graduate high school	1	0.9
High school degree	3	2.6
Some college	11	9.6
Associates/technical certificate	10	8.7
Bachelors	39	33.9
Maters	36	31.3
Doctoral degree	8	7
Economic hardship		
No difficulty paying bills	75	65.2
Do not expect to experience bad times	104	91.3
Do not expect to go without basic needs met	102	89.6
End up short on money at end of month	12	10.4
Child gender		
Female	128	55.7
Male	90	39.1
Childcare at least 5 h/week		
Yes	116	50.4
No	106	46.1
	*M*	Range
Child age	3y6mo	1y10m – 5y9mo

After participants registered for the public library program, they were invited to participate in the evaluation study by trained research staff. Using the email addresses from the registration list, caregivers were sent an email with an explanation of the evaluation and a link to the consent form and pre-survey, including demographic questions. Caregivers who had not completed the online pre-survey prior to the start of the program were invited to participate in-person before or immediately after the first session. Reminders were sent *via* email to caregivers who had not yet completed the survey after the first session. Caregivers who joined the program in Week 2 were able to complete a pre-survey at that session, but no one was asked to complete a pre-survey after Week 2 of the program. In the final week of the program, researchers distributed paper post-surveys in-person to all in attendance. Caregivers who did not complete a pre-survey were also asked to complete demographic items at the post-survey. Caregivers who were unable to complete the post-survey in person were sent an email request with a link to the online version of the survey.

### Measures

#### Program MS questionnaire

At pre-and post-test, caregivers completed a 16-item investigator-developed Math & Science Questionnaire: MSQ (Authors, unpublished), that captured beliefs (e.g., “Children learn best when they can explore a math and science concept with their five senses, rather than being directly told about the concept.”) and behaviors (e.g., “I use everyday opportunities to incorporate math and science concepts into daily routines with my child.”) for supporting young children’s math and science learning. Caregivers reported on each item on a 5-point scale from 1 (*Strongly disagree*) to 5(*Strongly agree*). Each item was examined as an independent outcome. Table 2 provides each of the program outcomes with an abbreviation for use in the remaining tables.

**Table 2:** Program specific questions and abbreviations.

Full item	Abbreviation
The library is a place I can go to learn about how to be a better parent/caregiver.	Library for caregiving
As a parent/caregiver, I play an important role in my child’s math and science education.	Important role in MS
Children learn important math and science concepts before entering kindergarten.	Learn MS before K
It is difficult for parents/caregivers to find opportunities at home to help children develop scientific and mathematical skills.	Difficult for MS at home^a^
Young children learn math and science concepts best through play, rather than in structured environments.	Learn MS through play
It is more important to praise children for getting the correct answer than to praise them for the effort or process it took to arrive at that answer.	Outcome-based praise^a^
It is sometimes better to just tell young children the answer to a question instead of giving children hints or asking questions so they figure out the answer on their own.	Tell children answer^a^
Children learn best when they can explore a math and science concept with their five senses, rather than being directly told about the concept.	Explore MS through senses
I tend to ask my child more close-ended questions (e.g., “What letter am I pointing to?”) than open-ended questions (e.g., “What do you think will happen if…?”).	Ask close-ended questions^a^
I frequently ask “why” questions to encourage my child to explain their way of thinking about a question.	Ask “why” questions
I use everyday opportunities to incorporate math and science concepts into daily routines with my child.	MS in daily routines
When playing with my child, I typically decide how the activity will go instead of following my child’s lead.	Parent-led activities
I feel comfortable talking to other parents about my child’s development.	Comfort talking to other parents
I have regular opportunities to interact with other parents	Interact with other parents
I feel prepared to support my child’s math and science education.	Prepared to support MS

^a^Indicates negatively valanced program items, where decreases from pre-to post-test are expected.

#### Parenting behaviors

Caregivers reported on their parenting behavior using the Raising Children Checklist (RCC; Shumow et al., 1998). The RCC includes three subscales: Firm (5 items; e.g., “Do you try to explain the reasons for the rules that you make?”; α_pre_ = 0.59, α_post_ = 0.54), Harsh (5 items; e.g., “Do you expect your child to obey you without any questions asked?”; α_pre_ = 0.60, α_post_ = 0.72) and Permissive (5 items; e.g., “Do you let your child decide what his/her schedule will be?”; α_pre_ = 0.70, α_post_ = 0.62).

#### Parental self-efficacy

Caregivers reported on their self-efficacy using the Parental Self-Agency Measure (Dumka et al., 1996). The scale contains 5 items (e.g., “I know I’m doing a good job as a parent.”; *α*_pre_ = 0.76, *α*_post_ = 0.79) measured on a 5-point scale ranging from 1 (*Almost never or never*) to 5(*Almost always or always*).

#### Progressive parenting

Caregivers reported on the extent to which they endorsed progressive parenting beliefs using the Progressive subscale of the Parental Modernity Scale (Schaefer and Edgerton, 1985). The scale includes 8 items (e.g., “It’s all right for a child to disagree with his/her parents”; *α*_pre_ = 0.64, *α*_post_ = 0.73) measured on a 5-point scale ranging from 1 (*Strongly disagree*) to 5 (*Strongly agree*).

#### Covariates

Caregivers reported on their child’s age and sex (0 = female, 1 = male) Caregivers also reported on their educational level (1 = 8th grade or less, 9 = PhD, MD, JD) and their race/ethnicity. Due to small numbers of racial/ethnic sub-groups, we examined differences between White (coded as 0) and Non-White (coded as 1) participants.

### Analytic plan

A static-group comparison design was employed. We compared whether the post-test scores for caregivers who completed the FMS program in the fall/winter (coded as 0) differed from the pre-test scores for caregivers who completed the FMS program in the spring (coded as 1). We controlled for child and family characteristics, including child age, sex, caregiver level of education, and child minority status. Analyses were conducted using Stata using full information maximum likelihood to handle missing data, which minimizes bias in parameter estimates while retaining the original sample size (Enders, 2010). There were no significant differences in any demographic characteristics or program outcomes at pre-test between fall/winter and spring participants.

## Results

Descriptive statistics demonstrated promising trends, with fall/winter participants reporting higher average levels of positive caregiving outcomes at post-test than spring participants at pre-test (see Table 3). Regression analyses compared post-survey results from fall and winter participants to pre-survey results from spring participants (see Table 4). Significant differences were found for three program-specific outcomes in the expected direction. At post-test, fall/winter caregivers felt 36-SD more prepared to support their child’s math and science education (*B* = 0.65, *SE* = 0.14, *β* = 0.36, *p* < 0.001), were 0.11-SD more likely to ask “why” questions (*B* = 0.16, *SE* = 0.06, *β* = 0.11, *p* = 0.01), and were 0.26-SD less likely to utilize parent-as opposed to child-directed play (*B* = −0.56, *SE* = 0.22, *β* = −0.26, *p* = 0.01), as compared to the spring caregivers at pre-test. Significant differences did not emerge on program-specific items related to caregivers’ beliefs regarding children’s math and science learning. In addition, no significant differences were found regarding caregiving more generally (i.e., parenting behaviors, parenting self-efficacy, or progressive parenting beliefs). Covariates also indicated significant differences in program outcomes, including general caregiving beliefs and styles, and attitudes, knowledge, and behavior regarding math and science (see Table 4 for full results).

**Table 3:** Descriptive statistics and correlations for study variables (Spring pre-test *n* = 35, Fall/winter post-test *n* = 80).

Outcomes	Time	*M*	*SD*	Min	Max	Covariates			
						Parent Ed.	Race^b^	Gender^c^	Age
						*r*	*r*	*r*	*r*
Library for caregiving	**S Pre**	4.21	0.72	2	5	−0.35	0.06	0.10	−0.18
	**F/W Post**	4.26	0.81	1	5	0.34*	−0.02	−0.08	0.07
Important role in MS	**S Pre**	4.63	0.49	4	5	−0.31	0.12	0.02	−0.96*
	**F/W Post**	4.81	0.62	1	5	0.55**	0.16	0.10	0.10
Learn MS before K	**S Pre** ^+^	4.65	0.57	3	5	−0.11	0.22	0.07	−0.95^+^
	**F/W Post**	4.79	0.63	1	5	0.40**	0.26	0.16	−0.02
Difficult for MS at home ^a^	**S Pre** ^+^	2.75	1.36	1	5	0.39^+^	−0.32	−0.02	0.91*
	**F/W Post**	2.29	1.27	1	5	0.01	−0.37*	0.25	0.27
Learn MS through play	**S Pre**	4.04	0.82	2	5	0.03	0.00	0.00	0.16
	**F/W Post**	4.38	0.89	1	5	−0.01	0.24	0.17	−0.05
Outcome-based praise ^a^	**S Pre**	2.50	1.38	1	5	0.01	−0.22	−0.06	0.49
	**F/W Post**	2.51	1.38	1	5	−0.32*	−0.47**	0.19	0.14
Tell children answer ^a^	**S Pre**	2.04	1.16	1	5	0.05	−0.25	0.04	0.06
	**F/W Post** ^+^	1.79	0.98	1	5	−0.07	−0.27	0.00	0.28^+^
Explore MS through senses	**S Pre**	4.38	0.65	3	5	−0.16	0.07	−0.23	−0.45
	**F/W Post**	4.56	0.83	1	5	−0.09	−0.04	−0.02	−0.10
Ask close-ended questions ^a^	**S Pre**	3.29	1.16	1	5	0.01	−0.62**	0.29	−0.11
	**F/W Post**	2.98	1.13	1	5	−0.13	−0.23	0.18	0.11
Ask “why” questions	**S Pre**	4.00	0.78	2	5	−0.23	0.00	−0.17	–
	**F/W Post**	4.15	0.64	3	5	−0.12	−0.07	−0.14	−0.13
MS in daily routines	**S Pre**	3.96	0.91	2	5	−0.33	−0.12	0.10	−0.65
	**F/W Post**	4.33	0.55	3	5	−0.16	0.11	0.12	−0.12
Parent-led activities ^a^	**S Pre**	2.96	1.20	1	5	0.15	−0.53*	0.04	0.41
	**F/W Post**	2.35	1.09	1	5	−0.09	−0.48**	0.00	−0.01
Comfort talking to other parents	**S Pre**	4.25	0.79	2	5	−0.25	0.10	−0.03	0.16
	**F/W Post**	3.96	0.81	1	5	0.01	−0.02	0.17	0.06
Interact with other parents	**S Pre**	3.88	1.15	1	5	−0.40	−0.15	0.04	−0.38
	**F/W Post**	3.96	0.88	2	5	0.02	0.17	−0.12	−0.04
Prepared to support MS	**S Pre**	4.00	1.10	2	5	0.12	0.06	−0.04	0.38
	**F/W Post**	4.57	0.57	3	5	0.10	0.14	0.17	0.00
Harsh ^a^	**S Pre**	2.10	0.62	1.25	3.80	0.21	−0.29	0.14	0.57
	**F/W Post**	2.08	0.52	1.20	3.60	−0.33*	−0.36*	0.29*	0.13
Firm	**S Pre**	3.50	0.39	2.80	4.00	−0.22	0.31	−0.12	−0.15
	**F/W Post**	3.58	0.33	3.00	4.00	−0.16	−0.12	−0.14	−0.26
Lax ^a^	**S Pre**	1.82	0.57	1.00	3.00	0.24	−0.23	−0.16	0.82
	**F/W Post**	1.74	0.45	1.00	3.00	0.20	−0.18	−0.18	0.26
Progressive parenting	**S Pre**	30.74	4.14	24.00	39.00	−0.04	0.59**	−0.05	−0.03
	**F/W Post**	31.98	4.23	16.00	38.00	0.41**	0.29	−0.19	0.01
Parental self-efficacy	**S Pre**	3.97	0.49	2.80	4.80	−0.03	0.17	0.14	−0.20
	**F/W Post** ^+^	3.97	0.51	2.80	5.00	−0.21	−0.16^+^	−0.09	−0.25
					***M***	6.90	0.41	0.70	3.58
					***SD***	1.57	0.49	0.46	0.83
					**Min**	1	0	0	1.87
					**Max**	9	1	1	5.76

S pre, Spring, Pre-test; F/W Post, Fall/Winter Post-test, ^+^p < 0.10, *p < 0.05, **p < 0.01. ^a^Indicates negatively valanced program items, where decreases from pre-to post-test are expected, ^b^White = 0, ^c^male = 1.

**Table 4:** Changes in program and caregiving outcomes using pre-post design.

Program outcomes	Time (Spring Pre = 0)				Parent Ed				Race (White = 0)				Gender (Female = 0)				Age			
	B	SE	*p*	*β*	B	SE	*p*	*β*	B	SE	*p*	*β*	B	SE	*p*	*β*	B	SE	*p*	*β*
Library for caregiving	0.05	0.04	0.26	0.03	0.06	0.09	0.53	0.12	0.03	0.12	0.82	0.02	0.02	0.15	0.91	0.02	−0.05	0.27	0.86	−0.03
Important role in MS	0.10	0.07	0.18	0.08	0.14	0.11	0.21	0.36	0.05	0.11	0.65	0.04	**−0.17**	**0.03**	**0.00**	**−0.24**	0.11	0.15	0.44	0.09
Learn MS before K	0.02	0.07	0.78	0.01	0.12	0.11	0.27	0.30	**0.20**	**0.07**	**0.00**	**0.16**	**−0.22**	**0.05**	**0.00**	**−0.28**	0.21	0.15	0.18	0.17
Difficult for MS at home^a^	−0.19	0.16	0.25	−0.06	0.08	0.08	0.28	0.10	**−0.49**	**0.16**	**0.00**	**−0.18**	**0.76**	**0.04**	**0.00**	**0.46**	**0.44**	**0.03**	**0.00**	**0.17**
Learn MS through play	0.31	0.28	0.27	0.16	0.00	0.07	0.98	0.00	0.16	0.22	0.49	0.09	0.00	0.28	0.99	0.00	0.20	0.13	0.13	0.11
Outcome-based praise^a^	0.40	0.24	0.09	0.14	**−0.24**	**0.11**	**0.03**	**−0.28**	**−0.78**	**0.19**	**0.00**	**−0.29**	**0.60**	**0.20**	**0.00**	**0.35**	0.26	0.20	0.20	0.09
Tell children answer^a^	−0.11	0.29	0.70	−0.05	−0.06	0.06	0.31	−0.09	−0.34	0.21	0.12	−0.16	0.42	0.28	0.13	0.31	−0.06	0.32	0.84	−0.03
Explore MS through senses	0.15	0.15	0.30	0.09	−0.05	0.08	0.50	−0.10	0.08	0.19	0.68	0.05	−0.07	0.25	0.77	−0.07	−0.09	0.17	0.58	−0.06
Ask close-ended questions^a^	−0.26	0.18	0.14	−0.11	−0.05	0.07	0.51	−0.07	**−0.51**	**0.24**	**0.03**	**−0.23**	0.28	0.22	0.19	0.20	0.38	0.21	0.07	0.17
Ask “why” questions	**0.16**	**0.06**	**0.01**	**0.11**	−0.08	0.05	0.10	−0.20	0.03	0.22	0.91	0.02	**0.00**	**0.19**	**0.00**	**0.00**	**−0.16**	**0.07**	**0.04**	**−0.11**
MS in daily routines	0.28	0.16	0.08	0.19	−0.08	0.06	0.18	−0.18	0.06	0.12	0.63	0.04	−0.23	0.29	0.43	−0.27	**0.22**	**0.07**	**0.00**	**0.16**
Parent-led activities	**−0.63**	**0.22**	**0.01**	**−0.26**	0.04	0.10	0.69	0.05	**−0.73**	**0.29**	**0.01**	**−0.33**	0.16	0.30	0.59	0.11	−0.01	0.11	0.90	−0.01
Comfort talking to other parents	−0.32	0.24	0.18	−0.18	−0.08	0.09	0.39	−0.15	**0.24**	**0.10**	**0.01**	**0.15**	0.12	0.25	0.62	0.12	0.13	0.23	0.57	0.08
Interact with other parents	0.04	0.26	0.86	0.02	−0.12	0.06	0.05	−0.20	0.09	0.17	0.57	0.05	−0.03	0.12	0.82	−0.02	−0.17	0.21	0.42	−0.09
Prepared to support MS	**0.65**	**0.14**	**0.00**	**0.36**	0.04	0.10	0.68	0.08	0.14	0.15	0.35	0.09	0.14	0.37	0.70	0.13	0.11	0.18	0.54	0.06
General outcomes																				
Harsh parenting	0.05	0.09	0.53	0.07	**−0.03**	**0.01**	**0.00**	−0.13	0.02	0.09	0.84	0.02	**−0.10**	**0.04**	**0.02**	**−0.22**	−0.07	0.08	0.37	−0.10
Firm parenting	0.04	0.12	0.74	0.03	−0.05	0.04	0.21	−0.14	**−0.34**	**0.05**	**0.00**	**−0.30**	**0.16**	**0.06**	**0.00**	**0.23**	0.23	0.16	0.15	0.21
Lax parenting	0.03	0.08	0.74	0.03	0.04	0.03	0.21	0.13	**−0.22**	**0.09**	**0.01**	**−0.23**	**0.29**	**0.08**	**0.00**	**0.47**	−0.17	0.12	0.16	−0.17
Progressive parenting	1.00	0.61	0.10	0.11	0.67	0.41	0.11	0.26	**2.32**	**1.14**	**0.04**	**0.28**	−0.69	0.49	0.16	−0.13	**−0.73**	**0.31**	**0.02**	**−0.09**
Parental self-efficacy	−0.03	0.19	0.86	−0.03	−0.03	0.06	0.68	−0.08	−0.11	0.10	0.18	−0.11	−0.13	0.10	0.18	−0.22	0.00	0.07	0.99	0.00

Bold indicates significant findings, ^a^indicates negatively valanced program items, where decreases from pre-to post-test are expected.

## Discussion

This study provides initial evidence of the effectiveness of the FMS program in promoting caregiver involvement in children’s early MS learning. Results have implications for promoting young children’s school readiness in community spaces, such as public libraries. The static-group comparison findings (i.e., using different waves of data collection from one larger study to create a comparison group) heighten the rigor of the study compared to a traditional single group pre-post design, and present a model for other community programs with similar “real world” data collection constraints.

### Program outcomes

#### Math and science practices, knowledge, and beliefs

The results of the current study are promising for the FMS program to increase caregivers’ ability to support their child’s early math and science learning. In particular, the program outcomes for which fall/winter families demonstrated improvement at post-test compared to the spring families at pre-test were primarily those that centered on program specific behaviors that can be enacted in the home to support early math and science learning. In contrast, program specific outcomes that captured beliefs regarding math and science learning in early childhood did not demonstrate significant differences between the groups. Specifically, caregivers asked more “why” questions to encourage their child to explain their thinking and to take their child’s lead during activities. By enacting these behaviors, caregivers likely felt more prepared to support their child’s math and science education. Another parent education program, although conducted within the elementary school setting, also found that participating parents demonstrated both increases in the educational activities they engaged in with their children at home, as well as in their role as crucial supporters of their child’s learning (Chrispeels and Rivero, 2001).

The fact that significant differences were found regarding program specific behaviors and skills, but not beliefs, regarding science and math learning in early childhood (e.g., Children learn important math and science concepts before entering kindergarten), is consistent with the nature of FMS programming. Through book reading, songs, and interactive activities focused on specific math and science topics (e.g., counting, patterns, asking scientific questions), the program focuses on modeling and practicing concrete behaviors to improve early STEM skills and positive caregiver-child interactions. The FMS program also encourages caregivers to act as co-learners, rather than leaders in their children’s play. It is likely that caregivers, especially those who chose to attend FMS, already come into the program with strong beliefs regarding the importance of early math and science skills, but do not feel like they have concrete strategies to support this learning. This is consistent with previous research that has shown consistently high positive beliefs regarding the importance of mathematics for young children and the capacity for young children to learn math, but more variability in parents’ reported math practices in the home (Missall et al., 2015; Şahin Çakır and Uludağ, 2022). In addition, caregiving beliefs are deeply engrained and culturally informed (Sigel and McGillicuddy-De Lisi, 2002), and therefore harder to change. This likely requires a more intensive intervention beyond the scope of the six-week FMS program. However, the significant findings reported here are consistent with other enhanced storytime programs utilizing interactive parent–child activities allowing caregivers to practice new skills in real time (Stewart et al., 2014; Taylor et al., 2020).

#### General caregiving

It is important to note that we did not see changes in general caregiving practices or beliefs, including parenting style, progressive parenting, or parental self-agency. We expected that as caregivers learned more about child development and engaged in child-directed activities to support their child’s learning, they would be more likely to believe in the importance of supporting their children’s interests, providing choices, and explaining decisions and rules, as reflected in progressive beliefs and a firm parenting style, in addition to feeling more self-efficacious overall. However, these results indicate that the program may not include enough explicit content to support generalization beyond the specific math and science topics that were emphasized throughout the course. It is also likely that it takes time for caregivers to internalize these ideas and see changes in their children’s learning and development. It is certainly possible that if caregivers continued to engage in these behaviors at home beyond the six-week intervention, that they may see the connections between their caregiving more generally and child outcomes. Future program developers or implementers may need to be more intentional in discussing how practices related to supporting math and science learning can be integrated into other domains of caregiving. Changing fundamental practices and perspectives regarding caregiving more generally may require a more intensive and sustained intervention. Finally, as discussed further below, these practices and attitudes may not hold the same relevance across demographic, especially racial/ethnic, groups; careful consideration should be given to choosing program outcomes that are both aligned with the program content and goals, as well as the families whom the program is targeting.

### Demographic considerations

It is important to note that considerable demographic differences emerged related to our program outcomes, as indicated through the inclusion of our covariates. Unfortunately, we did not have adequate power to examine whether the library program had differential effectiveness according to such characteristics. However, examining mean-level differences in our outcomes can provide insight regarding how community-based programs, especially those focused on math and science, may best be able to uniquely support particular groups of children and families. This is especially important, as the majority of librarians are White females from middle-class backgrounds (Bourg, 2014; Gohr, 2017), but the families that libraries serve are diverse across a wide range of social identities.

In general, caregivers of boys, caregivers with older children, and caregivers who did not identify as White found it more difficult to find opportunities at home to help children develop scientific and mathematical skills. Additionally, caregivers with lower education levels, caregivers who do not identify as White, and caregivers of older children were more likely to endorse the importance of outcome-based, as compared to effort-based, praise. Racial/ethnic and gender differences also emerged regarding the extent to which caregivers engaged in child-directed play and asked open-ended questions. Each of these caregiving behaviors were taught through FMS tips and activities, so it is crucial to ensure that program content and materials are able to reach families from diverse backgrounds. For example, providing options for downward and upward extensions of program activities can allow families to adapt such activities to be most developmentally appropriate for their child (Klein et al., 2008). In addition, incorporating adequate gender and racial/ethnic/cultural representation in program materials can ensure that families feel like the program content is relevant to them and their home context (Lau, 2006); this can also be enhanced through the involvement of program facilitators who represent the backgrounds and identities of families and children and who are typically under-represented in early childhood spaces, including males and people of color (Phillips et al., 2016). Finally, attention needs to be given to ensuring that materials are accessible for caregivers with lower levels of education; this is especially the case for programs that focus on math and science learning, as these caregivers may feel less self-efficacious regarding math and science concepts themselves (Haylock, 2007). Ensuring the representativeness, accessibility, and cultural relevance of program materials and content will likely promote the increased effectiveness of the program for all families and children, especially those who face disparities at school entry.

In addition to program-specific outcomes, demographic differences also emerged according to parenting styles and beliefs (i.e., firm, harsh, lax, progressive parenting), with caregivers with higher education levels and younger children more likely to engage in firm parenting, and caregivers who do not identify as White and parents of older children more likely to engage in harsh and lax parenting. Caregivers of girls and caregivers who identified as White were also more likely to endorse progressive parenting. Previous research has also found similar differences in parenting style according to demographic characteristics (Okagaki and Frensch, 1998; Shumow et al., 1998; Iruka, 2009; Keels, 2009; Bornstein et al., 2011; Parent et al., 2011; Fasoli, 2014). Historically, firm parenting, whereby caregivers set and communicate clear expectations that children are able to internalize and achieve while also providing opportunities for child autonomy, has been associated with positive academic and behavioral adjustment for young children (Rinaldi and Howe, 2012; Pinquart, 2016); however, this literature has been critiqued for its overreliance of White, middle-to upper-class samples. Research with more diverse samples within the US and across the globe have called into question whether these parenting styles hold the same relevance across cultures, and whether firm parenting is as beneficial, and harsh/lax parenting as detrimental, for non-White, non-middle-class populations (see Pinquart and Kauser, 2018 for a meta-analysis). Therefore, while many parenting programs have aimed to increase firm and/or reduce harsh and lax parenting behaviors, consideration should also be given to the alignment between this aim and families’ cultural backgrounds and values that shape parenting styles. Increased scholarship and discourse has focused on promoting anti-racism and social justice within library services and programs (Espinal et al., 2018). One possible approach can be to ensure that program goals and outcomes do not assume White cultural values and beliefs as the norm (Stauffer, 2017).

### Lessons learned

This evaluation of the FMS program imparts two important lessons for practitioners and researchers working in community settings. Specifically, it provides guidance on the development and implementation of parent–child interactive programming, as well as a novel approach to program evaluation, moving beyond the traditional pre/post design.

First, our results emphasize the promise for parent education programs that involve the child in interactive activities for enhancing caregiving skills. By providing caregivers with opportunities within the sessions to practice the skills they were learning, we saw significant changes between our treatment and comparison groups on items that directly addressed practices related to children’s math and science learning. Considering the age of the participating children (i.e., preschool age), it was important to develop and successfully implement a program that both taught concrete caregiving information and skills, but was also engaging for young children. The interactive storytime format provided opportunities for didactic teaching of program content, book reading, and movement through song and dance, while still having a large block of time for unstructured play and activities to explore the focal math and science concept of the week. In addition, the group-based nature of the program allowed families and children to learn from one another, engage in parallel play, and build relationships over the course of the program. Future program developers should consider a variety of ways to structure programs that facilitate both caregiver and child engagement, as well as skill development.

Second, in our partnership with the public library that developed and implemented the FMS curriculum, the need to consider feasible evaluation strategies was apparent. Although RCTs are a gold standard approach for program evaluation, they are not always practical or possible in many community-based situations, especially without significant additional, and often external, resources. The focus on ensuring internal validity can limit external validity or ignore the realities of providing an accessible and flexible program to families. In addition, the time and resources necessary to conduct rigorous evaluations can be prohibitive, especially as public institutions, such as libraries, grapple with funding concerns or legislation that may impact the timeline for programming and evaluation. At the same time, it is important to recognize the limitations of less rigorous designs, such as pre-post examinations that cannot control for issues such as history or maturation. Employing more rigorous evaluation designs, such as the static group comparison presented here, that both meet the needs and reality of community-based organizations and reduce methodological concerns, is crucial to engage in participatory work between academic and public partners. In this case, because of the staggered delivery of the program, we were able to create a comparable control group within our sample, without limiting the families who received the program or the timeline for receiving it. Our approach is more rigorous than a typical static-group comparison that utilizes a general community sample for a comparison group, as they likely differ across a variety of characteristics, most importantly their desire to participate in the program. Various research designs exist that can be employed to meet the needs of community organizations and researchers implementing and evaluating a wide array of programs (Shadish et al., 2001). To do so, all partners must understand both the needs and realities of the community setting and the population it serves, as well as the expectations for conducting research in that setting to learn about the potential effectiveness of the program on identified outcomes.

### Limitations

Although this study provides important information regarding the effectiveness of the FMS program for caregiving behavior, it is not without limitations. First, our study sample was relatively small to begin with and was even smaller after using one of the time-points of data as a comparison group. As a result, the study has low power, increasing the likelihood of undetected or under-detected effects. In addition, our findings may be limited by selection bias, as parents who knew about and elected to enroll in the program may score higher on some of the practices measured than the general population. Therefore, it is encouraging that significant results did emerge; however future studies should replicate these findings with a larger sample of families, including those who may not have known about the program on their own. As a result, all findings presented here are exploratory in nature and should be interpreted conservatively.

Second, the participants in the study were relatively homogenous, especially in terms of gender and education-level; results may not generalize to male caregivers, families with low levels of education, or racial/ethnic groups not well represented in the study (e.g., Black, Native American families). Future work to evaluate the effectiveness of this program would benefit from recruiting a more diverse community sample of parents and young children including those who do not frequent the library setting. This may be achieved by ensuring that library-based programs, such as FMS, actively engage in anti-racist and equity-explicit approaches in both content (e.g., incorporating materials that reflect the background and values of families) and delivery (e.g., minimizing barriers to participation).

Third, the data collected were all parent-report and the Math & Science Questionnaire used to capture program specific beliefs and practices is an investigator-developed measure used here for the first time. While this measure showed high internal consistency and predictive validity, its convergent and discriminant validity have not been tested. Future studies would benefit from the use of multi-method assessments of caregiving and need to examine the relationship between scores on the MSQ and well-established measures of positive caregiving to confirm its overall validity.

Finally, although we believe the static-group comparison research design added methodological rigor to the study (compared to a traditional pre-post design), it is not without limitations. Due to the correlational nature of the study, this evaluation was unable to isolate program impact. Therefore, a more rigorous experimental or quasi-experimental design to evaluate program effectiveness is needed. Despite the limitations associated with utilizing a post-test only design, strengths of the present study were the inclusion of covariates (i.e., parent education, race/ethnicity, child age, child gender) and a novel analytic approach to analyzing the data. As a result, the results are stronger than traditional mean comparisons (e.g., t-tests) because the estimates take into account outside factors that may be associated with caregiving outcomes and create a comparable control group.

### Conclusion

Despite the above limitations, the present study contributes novel findings regarding the promise of providing authentic STEM learning within community settings, through an enhanced storytime format. We find preliminary evidence that FMS can impact concrete caregiving behaviors related to early math and science learning. Due to its openness and accessibility, the library provides an exceptional opportunity for families to engage in programs to support their young children’s math and science learning. However, most of the families that participated in the program were White and of middle-high socio-economic status, so attention should be given to techniques that could reduce systemic barriers that might prevent families from participating in library programs and that may enhance alignment between families’ backgrounds and values with the program content and goals. Additionally, the nature of library programming also presents challenges for conducting a gold-standard rigorous evaluation; this is a concern likely shared by program implementers and evaluators in other community settings. Therefore, this paper emphasizes how other research designs – in this case, the static-group comparison design – can meet the needs of community-based programs and their participants, while still increasing the methodological rigor beyond a typical pre-post evaluation.

## Data availability statement

The raw data supporting the conclusions of this article will be made available by the authors, without undue reservation.

## Ethics statement

The studies involving human participants were reviewed and approved by the Arizona State University Institutional Review Board. The patients/participants provided their written informed consent to participate in this study.

## Author contributions

LG, MT, MP, and MW: study conception, design, and draft manuscript preparation. LG, MT, and MP: data collection, analysis, and interpretation of the results. All authors contributed to the article and approved the submitted version.

## Funding

This project was supported by the Arizona State Library, Archives & Public Records, a division of the Secretary of State, with federal funds from the Institute of Museum and Library Services.

## Conflict of interest

The authors declare that the research was conducted in the absence of any commercial or financial relationships that could be construed as a potential conflict of interest.

## Publisher’s note

All claims expressed in this article are solely those of the authors and do not necessarily represent those of their affiliated organizations, or those of the publisher, the editors and the reviewers. Any product that may be evaluated in this article, or claim that may be made by its manufacturer, is not guaranteed or endorsed by the publisher.
